# Epigenetic determinants of fusion-driven sarcomas: paradigms and challenges

**DOI:** 10.3389/fcell.2024.1416946

**Published:** 2024-06-14

**Authors:** Benjamin Z. Stanton, Silvia Pomella

**Affiliations:** ^1^ Nationwide Children’s Hospital, Center for Childhood Cancer Research, Columbus, OH, United States; ^2^ Department of Pediatrics, The Ohio State University College of Medicine, Columbus, OH, United States; ^3^ Department of Biological Chemistry and Pharmacology, The Ohio State University College of Medicine, Columbus, OH, United States; ^4^ Department of Hematology and Oncology, Cell and Gene Therapy, Bambino Gesù Children’s Hospital, IRCCS, Rome, Italy; ^5^ Department of Clinical Sciences and Translational Medicine, University of Rome Tor Vergata, Rome, Italy

**Keywords:** epigenetics, sarcoma, genomics, etiology, architecture

## Abstract

We describe exciting recent advances in fusion-driven sarcoma etiology, from an epigenetics perspective. By exploring the current state of the field, we identify and describe the central mechanisms that determine sarcomagenesis. Further, we discuss seminal studies in translational genomics, which enabled epigenetic characterization of fusion-driven sarcomas. Important context for epigenetic mechanisms include, but are not limited to, cell cycle and metabolism, core regulatory circuitry, 3-dimensional chromatin architectural dysregulation, integration with ATP-dependent chromatin remodeling, and translational animal modeling. Paradoxically, while the genetic requirements for oncogenic transformation are highly specific for the fusion partners, the epigenetic mechanisms we as a community have uncovered are categorically very broad. This dichotomy prompts the question of whether the investigation of rare disease epigenomics should prioritize studying individual cell populations, thereby examining whether the mechanisms of chromatin dysregulation are specific to a particular tumor. We review recent advances focusing on rhabdomyosarcoma, synovial sarcoma, alveolar soft part sarcoma, clear cell sarcoma, undifferentiated round cell sarcoma, Ewing sarcoma, myxoid/round liposarcoma, epithelioid hemangioendothelioma and desmoplastic round cell tumor. The growing number of groundbreaking discoveries in the field, motivated us to anticipate further exciting advances in the area of mechanistic epigenomics and direct targeting of fusion transcription factors in the years ahead.

## 1 Introduction

Of the 1.9 million cases of cancer each year in the United States, approximately 1 percent will be diagnosed with a sarcoma (Sarcoma Foundation of America; American Cancer Society). Despite the relative rarity of sarcoma in the broader context of human malignancy, the survival rates for sarcomas, and specifically fusion-driven subtypes of sarcoma, are dismal and have not improved dramatically in several decades (Shern et al., 2021). Of note, focusing on pathological germline variants within subtypes may even provide further resolution to these clinical outcomes (Martin-Giacalone et al., 2024). While fusion-driven sarcomas represent an overall minority in cancer diagnoses, the alarmingly low survival rates, the high likelihood of metastatic events, and the overall lack of progression of clinically promising molecules from the benchtop to the bedside has captured the attention of a broad spectrum of research teams from diverse backgrounds.

Excitingly, new insights have emerged in recent years that have catalyzed new context for the way we think about sarcomagenesis. We highlight several of these insights here. Despite “quieter” genomes in sarcoma (lacking high mutation rates), there has been recent compelling evidence that structural variation (SV) is much more common in sarcoma than previously thought, including but not limited to the definitional translocation events (Chen et al., 2015; Gryder et al., 2020a; Nacev et al., 2022; Shukla et al., 2022; Xu et al., 2022; Choo et al., 2023; Wang et al., 2023). We anticipate exciting advances in the coming years of deeper characterization of SV in diverse sarcomas, enabled in part by emerging innovative sequencing technologies and platforms.

Next-generation sequencing technologies have been highly impactful for characterization of SV events, and copy number variation (CNV) in sarcomas, and sequencing at the clinical level has been immensely impactful for diagnosis and characterization. We describe recent advances in the clinical genomics field, with a special focus on fusion-driven sarcomas. Given that many sarcomas have a general dearth of mechanistic etiology, the use of clinical genomics as an entry point for diagnosis, and in a “reverse translational” sense, using genomics classification as a driver to formulate mechanistic hypotheses, has been of immense impact.

We also note that a major area of focus in the mechanistic literature on sarcoma molecular etiology has been on establishing and mapping the core regulatory circuitry (CRC) in tumors, a mechanistic concept which is versatile and generalizable to diverse human cancers, stemming from initial reports in pluripotent tissues (Boyer et al., 2005). We highlight recent advances in understanding CRCs of fusion-driven sarcomas, with connections to clinical genomics, and translational epigenetics. Enabling maintenance of CRCs in fusion-driven sarcomas are the molecular motors, including ATP-dependent remodelers, which integrate the circuit. We discuss exciting recent advances connecting SWI/SNF-family remodelers with fundamental etiologic mechanisms in sarcoma.

**FIGURE 1:**
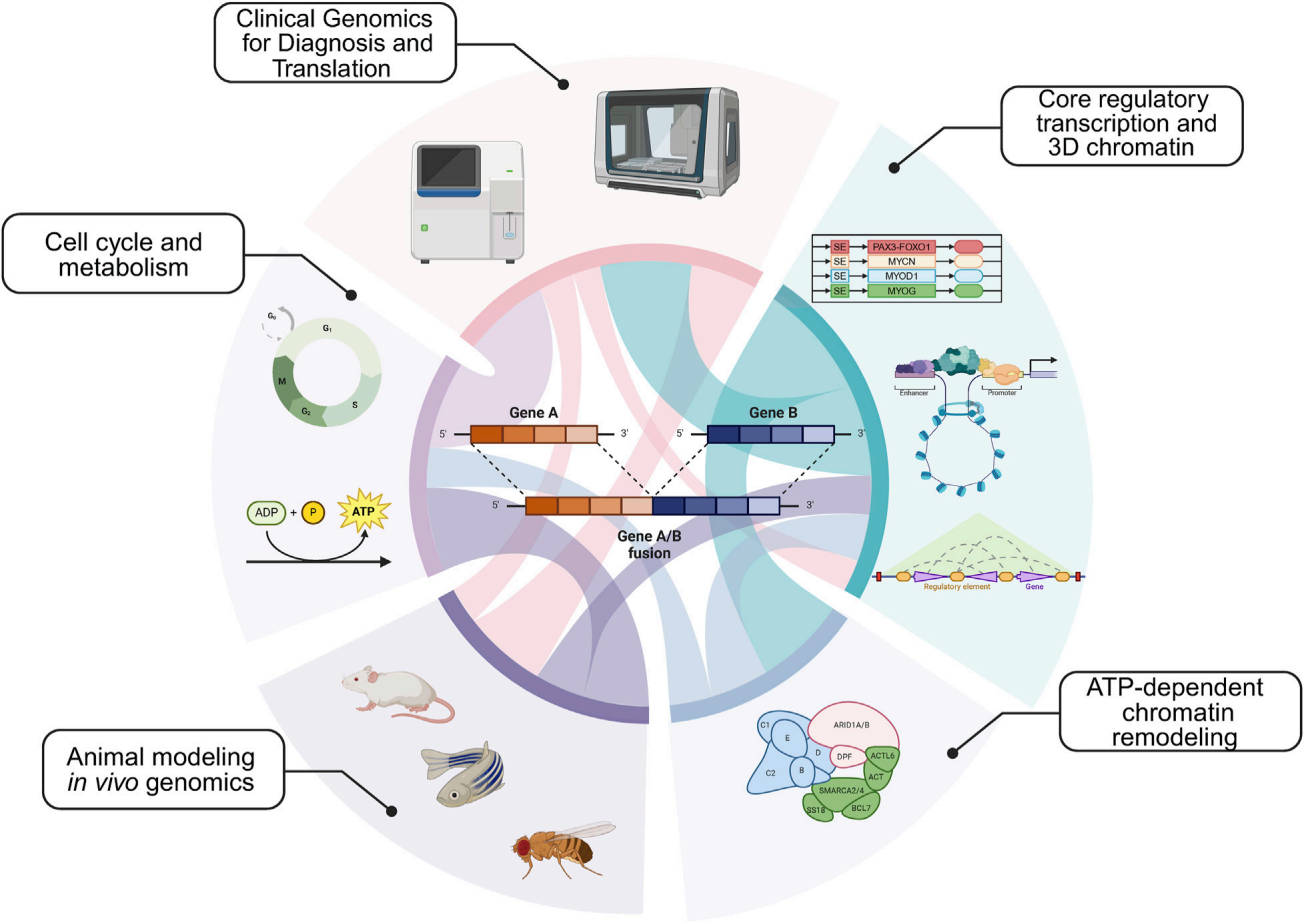
Major epigenetic themes in sarcoma etiology: Core regulatory circuitries (CRCs) and 3-dimensionalizing the core regulatory transcription; ATP-dependent remodeling complexes, including SWI/SNF; Animal modeling and *in vivo* gene targeting; Understanding the cell cycle and metabolism; Clinical genomics for diagnosis

**FIGURE 2:**
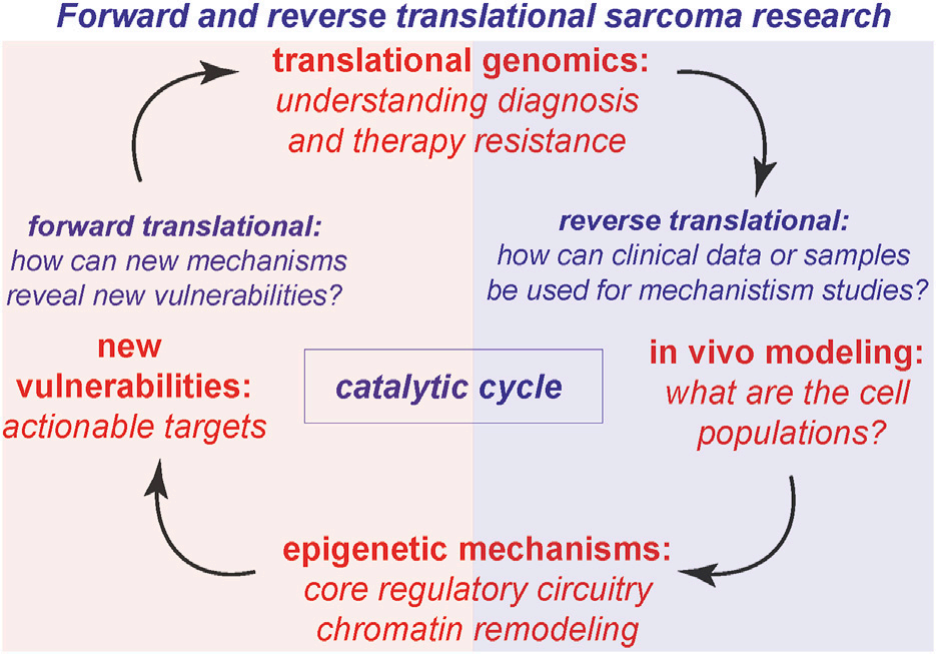
Reverse translational and forward translational science. The virtuous cycle of discovery, where translational genomics leads to modeling and new mechanisms, which reveal new vulnerabilities to catalyze forward translational impact.

An additional area of insight is understanding tumor proliferation in animal models for sarcomas. *How is the cell cycle regulated to maintain tumor proliferation in vivo? What types of sarcoma cell populations exist in cell lines versus animal models?* We address these key questions in the context of stimulating recent advances. In addition to the key areas above (Figure 1), we present our views on the next intellectual and mechanistic frontiers for mechanistic sarcoma research, both from the forward translational, and reverse translational perspectives. In so far as new exciting clinical data can inform basic mechanistic research, and new mechanisms can in turn illuminate actionable vulnerabilities, this virtuous cycle (Figure 2) is the subject of our outlook.

### 1.1 Clinical genomics for diagnosis and translation

A great mentor once said that “genetics always comes before biochemistry.” In this fundamental sense of the logical flow of scientific discovery, it is also critical that we understand the genetic landscape of tumors, before we initiate chromatin structural studies. The logical flow would be lacking if our research teams went from diagnosis, pathology, FISH to validate fusion status, straight into functional epigenetics. The idea that a tumor’s genomic landscape can be understood has been enabled conceptually from the sequencing of the first animal genome (Consortium, 1998) and from the sequencing of the first tumor genomes (Ley et al., 2008). Moreover, comparisons to tumor *versus* normal have been impactful since the initial strategy was reported (Ley et al., 2008). The concept of clinical genomics has been gaining momentum in the literature, especially with advances in bioinformatics technology and steadily decreasing costs associated with sequencing a tumor genome. Navigating the challenges and recognizing the significance of accurate diagnosis and clinical genomics are intertwined endeavors. The intricacies of interpreting genomic data and classifying variants present formidable obstacles, compounded by concerns such as data privacy and the demand for specialized expertise. Yet, the vital role of precise diagnosis and clinical genomics cannot be overstated. These practices enable the delivery of personalized medicine, tailoring treatment strategies to an individual’s genetic profile, thereby optimizing patient outcomes and minimizing adverse effects. Moreover, clinical genomics facilitates early detection of genetic disorders, empowering proactive interventions and enhancing public health outcomes. Despite the complexities, the integration of clinical genomics stands poised to revolutionize healthcare delivery. We describe recent efforts in clinical genomics and translational genomics with a focus on fusion-driven sarcomas.

**FIGURE 3:**
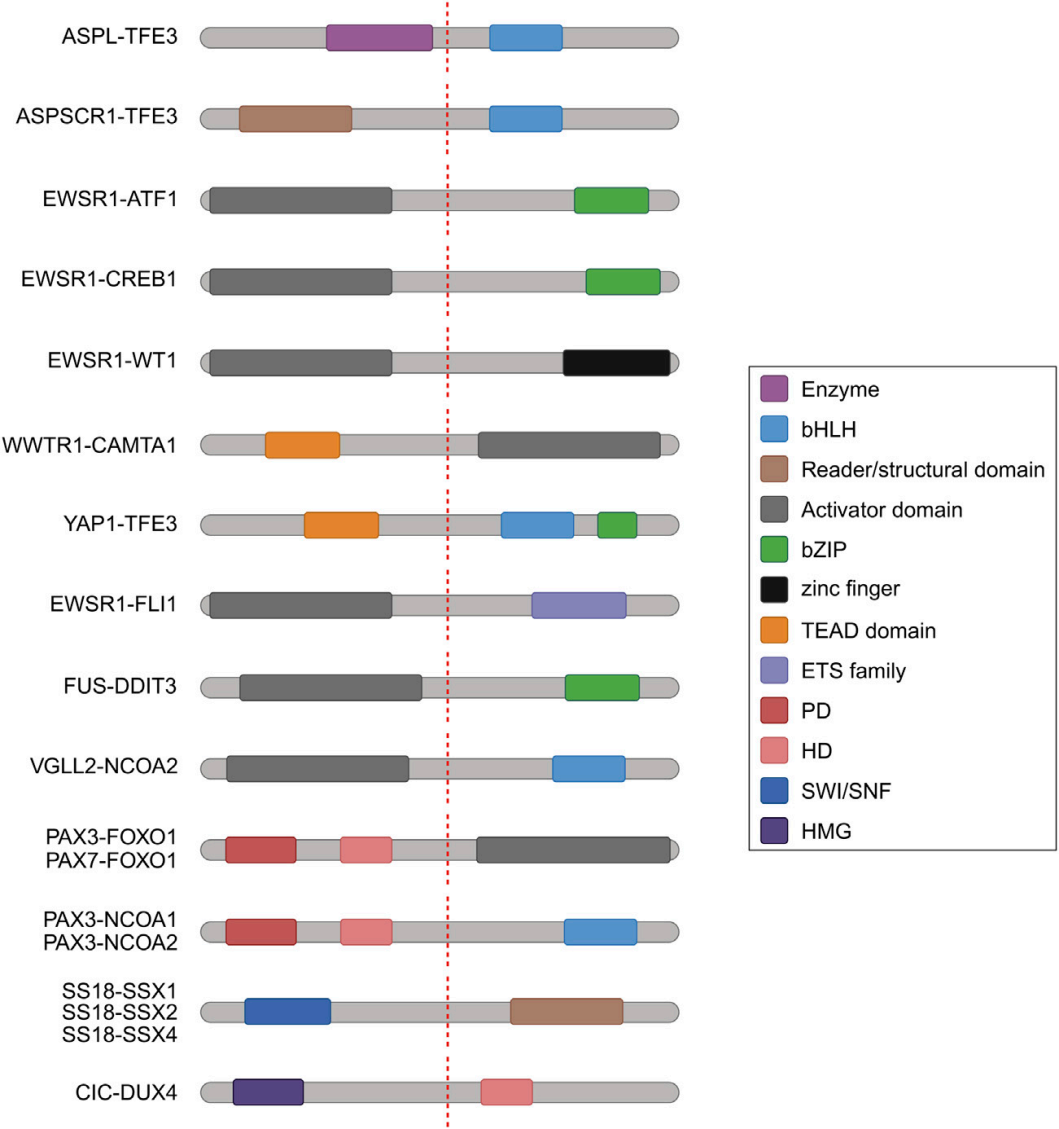
Schematic representation of domain architecture of fusion proteins. Domains and classes of fusion proteins are defined through color coding. Breakpoints are represented with dashed red lines. Abbreviations: bHLH, basic helix–loop–helix; bZIP, basic Leucine Zipper Domain; PD, Paired box domain; HD, homeobox domain; HMG, high-mobility group.

In diverse tumor types including Ewing sarcoma (ES), Desmoplastic Small Round Cell tumor (DSRCT), and Clear cell sarcoma (CCS), EWSR1 is expressed as a fusion transcription factor resulting in distinct morphologies and tissue lineages (Table 1; Figure 3). A recent study integrated standard histopathology, fusion-gene sequencing panels, and RNA sequencing for 13 cases to narrow the diagnostic focus through clinical genomics for EWSR1 fusions (Argani et al., 2020). Highlighting the impact of clinical genomics, from the set of clinical samples with EWSR1-CREB1 fusion events, subsets with distinct gene expression patterns and morphology, could inform narrower sub-type classifications for CCS (Argani et al., 2020). In a related study of 39 cases, sub-classification based on DNA methylation status was highly accurate in terms of predictive clustering of EWSR1-CREB, and related fusions as CSS versus other tumor types (Dermawan et al., 2022a). This is especially important because classification based on fusion status alone might not have differentiated between Angiomatoid fibrous histiocytoma (AFH) and CCS. This is reminiscent of key developments in neurooncology, where methylation classifiers are now more sensitive and predictive than RNA sequencing for diagnoses (Capper et al., 2018).

**TABLE 1:** The combinatorial complexity of domain architecture in fusion-driven sarcoma.

DNA-binding domain	Fusion partner	Malignancy
TFE3	ASPL	Alveolar Soft Part Sarcoma
TFE3	ASPSCR1	Alveolar Soft Part Sarcoma
ATF1	EWSR1	Clear cell sarcoma
CREB1	EWSR1	Clear cell sarcoma
WT1	EWSR1	Desmoplastic round cell tumor
CAMTA1	WWTR1	Epithelioid hemangioendothelioma
YAP1	TFE3*	Epithelioid hemangioendothelioma
FLI	EWSR1	Ewing Sarcoma
DDIT3	FUS	Myxoid/round liposarcoma
NCOA2	VGLL2	Rhabdomyosarcoma
PAX3	FOXO1	Rhabdomyosarcoma
PAX7	FOXO1	Rhabdomyosarcoma
PAX3	NCOA1**	Rhabdomyosarcoma
PAX3	NCOA2**	Rhabdomyosarcoma
SS18	SSX1, SSX2, SSX4	Synovial Sarcoma
DUX4	CIC**	Undifferentiated round cell sarcoma

Domains and classes are defined through color coding (columns 1, 2) with driver function in malignancies (column 3). The (**) denotes fusion partner is also DBD. In the DNA-binding domain (DBD) column, bHLH , PD , HD , bZIP/leucine zipper , zinc finger, TEAD domain , ETS family , and SWI/SNF are color coded in the text. In the Fusion partner column, Enzyme , Reader/structural domain , Activator domain , and HMG are color coded in the text.

Integrations of clinical genomics, pathology and FISH have revealed that EWSR1-fusions with the CREB family member ATF1 (EWSR1-ATF1) are also drivers in malignant mesothelioma (MM), in addition to CCS (Table 1; Figure 3) (Desmeules et al., 2017). EWSR1 fusions with YY1 (EWSR1-YY1) are also found in MM, and methylation profiling of these tumors results in clustering together with EWSR1-ATF1 fusions, despite having distinct clustering from a variety of other adult and pediatric tumors (Dermawan et al., 2022b). Recent integrations of clinical genomics with pathology and RNA sequencing have also enabled characterization of ALK-fusion drivers in MM, including TPM1-ALK and STRN-ALK (Argani et al., 2021).

In DSRCT, EWSR1 is fused with WT1, resulting in the expression of the EWSR1-WT1 chimeric transcription factor (Table 1; Figure 3) (Gedminas et al., 2022). Recent translational genomics efforts have uncovered that trabectedin analogues (lurbinectedin) can alter DNA binding preferences of the EWSR1-WT1 fusion, resulting in altered localization (Gedminas et al., 2022). In depth clinical genomics of DSRCT has uncovered a panel of actionable vulnerabilities in the tumor, or transcripts that are highly expressed and also are associated with clinically promising molecules (Hingorani et al., 2020).

With distinct bioinformatics approaches used across labs, there is an increasing need in the community for data sharing, especially in the context of rare disease where sample access can be challenging. We highlight key studies that have enabled high depth clinical genomics and data sharing across institutions. Key studies have emerged that highlight mutational signatures from large cohorts in rare disease, leveraging impact from statistical power. Molecular analysis of 17 Epithelioid hemangioendothelioma (EHE) patient samples by FISH identified the presence of the WWTR1-CAMTA1 fusion as useful for the correct diagnosis of EHE malignant tumors over epithelioid hemangioma, a benign tumor usually misdiagnosed due to overlapping histology (Errani et al., 2011). Furthermore, next-generation sequencing data within a multicenter and cross-sectional study including 49 EHE patient samples, revealed that 57.1% of participants harbor a secondary genomic variant in addition to the WWTR1-CAMTA1 fusion (Seligson et al., 2019). Intriguingly, the deletion of the *CDKN2A/B* locus, coding for known tumor suppressor genes, has been ranked as the most frequent genomic alteration, suggesting a potential role of *CDKN2A/B* in the development of EHE.

From a cohort of 299 ES patients, clinical genomics revealed recurrent mutations in *STAG2* and *TP53* (Tirode et al., 2014). A clinical genomics study with 147 matched rhabdomyosarcoma (RMS) and tumor normal samples revealed new insights into how signaling alterations in PI3K and *FGFR4* may synergize with altered in epigenetic modifiers (*PAX*-fusions, MYCN) to reinforce oncogenic gene expression (Shern et al., 2014). Recent studies have also seen an increase in statistical power and expanded sample cohorts. In a new study with data analysis harmonization across 2,138 bone and connective tissue sarcomas, the commonest alterations were associated again with the PI3K signaling axis and *TP53* (Nacev et al., 2022).

Up-to-date studies with a focus on RMS have revealed key attributes within and across patient samples both in the context of mutational signatures, and also in sub-populations that are active within a heterogenous tumor. In a study with 641 RMS patient samples, researchers harmonized across datasets to reveal key patterns in gene expression, survival and fusion status. Of note, secondary mutations were characterized across RMS subtypes, and were further classified based on anatomic location and survival. A key example from this work is that altered TP53 co-occurrence with *PAX3-FOXO1* fusion positive RMS was universally fatal (Shern et al., 2021). Understanding the functional genomics of RMS has been studied recently through a large multi-institutional collaboration and revealed four universal cell populations that systematically reside within RMS tumors (Danielli et al., 2023a). Through establishing a unanimous data analysis strategy, researchers were able to identify populations of cycling/proliferative, differentiated/skeletal muscle, mesenchymal/progenitor, and ground state cells across the spectrum of 72 analyzed samples. The depth and power of these clinical genomics studies will enable researchers to uncover key mechanisms of sarcomagenesis in the coming years. Moreover, as we transition from examining linear genomic landscapes into the examination of 3-dimensional (3D) epigenomic landscapes (Gryder et al., 2020a; Wang et al., 2023; Kim et al., 2024), we will begin to integrate genetic and epigenetic mechanisms driving diverse sarcomas.

### 1.2 Cell cycle and metabolism

While cell cycle epigenetics fundamentally links to the central definition of epigenetics, or how a cell state is inherited, this is an area that is either understudied or unstudied in sarcoma biology. Thus, there is incredible potential to make a vast impact through understanding how an epigenetic state is heritable in a sarcoma cell, in a bulk cell context, or more excitingly, within discrete populations in single cells. Key studies in developmental systems in this area have been pioneered in recent years in the context of nascent histone acetylation (Popova et al., 2021; Lovejoy et al., 2023) and nucleosome turnover (Flury et al., 2023; Wenger et al., 2023), along with exciting innovative methodologies to map chromatin dynamics in newly replicated chromatin (Petryk et al., 2021; Stewart-Morgan and Groth, 2023). What key advances can be made through combining these high impact technologies with central questions in cell cycle epigenetics in sarcoma? Among other exciting areas, linking of cancer metabolism with chromatin level alterations will be of high impact (Chen et al., 2022). We focus now on key studies that have illuminated some of these central questions and processes in fusion-driven sarcoma.

In Alveolar soft part sarcoma (ASPS), the ASPL-TFE3 fusion oncoprotein is found to regulate the cell cycle through P21 function (Table 1; Figure 3) (Ishiguro and Yoshida, 2016). With rapid inducible systems, it was found that ASPL-TFE3 expression upregulates p21/WAF1 and also alters the cell cycle when induced in human cells. Surprisingly, the overexpression of ASPL-TFE3 results in increased cell populations in G2/M phases of the cell cycle, decreased Rb phosphorylation and growth suppression, which is perhaps contrary to what we might hypothesize for the molecular functions of a tumor oncogene. However, in the literature it has been noted that fusion oncoproteins have both growth suppressive and growth inducing functions, with noted toxicity early in development (Keller et al., 2004a; Keller et al., 2004b). Other studies have confirmed the exciting effects of ASPL-TFE3 on increasing the G2/M cell populations (Fang et al., 2021) and contributing to altered cell cycle progression. Interestingly, at the chromatin level, ASPL-TFE3 binds to many promoters of lysosomal genes (Fang et al., 2021). The expression of the ASPL-TFE3 protein contributes to altered tumor amino acid metabolism, suggesting that it functions in tumorigenesis through linking altered epigenetic promoter activation with functional adaptation to tumor microenvironments which may be more nutrient poor.

Another great example of linking epigenetic functions of fusion oncoproteins with tumor metabolism is in Myxoid liposarcoma (MLPS), where the FUS-DDIT3 chimera activates PI3K/AKT signaling to drive the cell cycle (Table 1; Figure 3) (Berthold et al., 2022). Interestingly, FUS-DDIT3 functional activation of PI3K/AKT results in increased Hippo/YAP1 activity, which presents a potentially unique vulnerability in MLPS. The FUS-DDIT3 fusion interacts with and colocalizes with YAP1 in MLPS, to regulate MYC gene signatures and disrupt tissue differentiation (Berthold et al., 2022). Further work to dissect the transcription factor cooperativity mechanisms between FUS-DDIT3 and YAP1 will be of immense impact.

### 1.3 Core regulatory transcription and 3D chromatin

Cell identity and cell state are tightly controlled by the activity of transcription factors (TFs) that temporally and spatially coordinate the transcriptional program. The existence of a TF feed-forward loop that reinforces gene expression has been demonstrated both in physiologic and pathologic conditions, and termed core regulatory circuitry (CRC) (Saint-Andre et al., 2016). The in-depth use of sequencing technology made it clear that the cellular identity/state within a tumor, and especially those fusion-driven cancers, rely on the expression and activity of CR TFs, that lock the cells into a proliferative state, thus representing oncogenic etiology (Durbin et al., 2018). Mechanistically, CR TFs by recruiting chromatin-erasers, readers and writers, can control the epigenetic modifications at clustered enhancers, to which chromatin machinery is engaged in 3D looping, reshaping the epigenome (Gryder et al., 2019a; Gryder et al., 2019b). Based on this conceptual model, which in many ways is an extension of Laurie Boyer’s initial report of CRC in pluriopotent tissue (Boyer et al., 2005), interesting questions emerge regarding order of events: *is 3D epigenome folding a cause or consequence of transcriptional activity? How do fusion-TFs play a role in CRCs? Do fusion-TFs need to bind to repressed chromatin, in order to prime or establish epigenetically open states required for CRC maintenance?* We describe here recent efforts to understand the CRC and the 3D landscape of fusion-driven sarcomas.

In fusion-positive (FP)-RMS, PAX3-FOXO1 can bind to closed chromatin to form local nucleosome depleted regions (Sunkel et al., 2021) to establish *de novo* clustered enhancers which are bound by CRC TFs (Table 1; Figure 3) (Gryder et al., 2019a). Interestingly, the compacted chromatin binding of PAX3-FOXO1 can be kinetically resolved and measured to illuminate precise steps in RMS chromatin activation (Sunkel et al., 2021). However, hyperacetylation disrupts localized CRC TF function (Gryder et al., 2019a). The spreading of histone acetylation, upon HDAC inhibition, leads to the disruption of 3D chromatin architecture, which can be precisely quantified by AQuA-HiChIP (Gryder et al., 2020a). To provide a chromatin domain context for the local functions of tumor-essential TFs in RMS, a newly reported comprehensive 3D chromatin analysis has uncovered the large chromatin compartments and domains in which these regulatory functions are executed (Wang et al., 2023). Therapeutic targeting of Ras pathway activity (Yohe et al., 2018), histone acetylation (Gryder et al., 2019a; Gryder et al., 2019b; Gryder et al., 2020a; Gryder et al., 2020b) and lysine demethylase inhibition (Kim et al., 2024) halt RMS transcriptional activity thus impairing RMS growth. Interestingly, new evidence is emerging that PAX3-FOXO1 and PAX7-FOXO1 fusion proteins in FP-RMS have distinct DNA binding preferences and divergent capabilities for chromatin activation, determining a selective cell context for tumorigenic processes (Manceau et al., 2022).

In Synovial Sarcoma (SS), characterized by the fusion of the SS18 gene to either SSX1, SSX2, or SSX4, the expression of FOXM1 associates with poor prognosis and correlates with cell-cycle genes (Table 1; Figure 3). Moreover, FOXM1 inhibition (Thiostrepton) or genetic downregulation impairs SS growth and increased Doxorubicin sensitivity (Maekawa et al., 2016). We anticipate continued exciting advances in SS CRC in the coming years. In MLPS, mapping the clustered enhancer landscape enables the identification of CRC dependencies on the protein fusion FUS-DDIT3 and BET-proteins (Table 1; Figure 3) (Chen et al., 2019). Indeed, FUS-DDIT3 functions concordantly with BET-proteins for sustaining a tumorigenic enhancer-driven gene expression, and their cooperative regulation makes MLPS vulnerable to BET protein targeting.

The expression of chimeric TFs, YAP-TFE3 and WWTR1-CAMTA1, in EHE, drives tumor initiation *in vivo*, through increasing the amount of transcriptionally active chromatin. In this context, chromatin activation can occur through the direct binding with Ada2a-containing acetyltransferase (ATAC), a conserved histone acetyltransferase complex (Merritt et al., 2021). Interestingly, the expression of WWTR1-CAMTA1 in normal endothelial cells leads to fusion-dependent activation of oncogene-induced senescence (OIS), genomic instability and replication stress due to hyper-transcription (Neil et al., 2023). In agreement with molecular data from patients, loss of *CDKN2A*, the most frequent mutation in EHE cases, circumvents OIS and growth arrest (Neil et al., 2023).

In ES, EWS-FLI has been described as a master regulator of chromatin reprogramming (Table 1; Figure 3) (Adane et al., 2021; Showpnil et al., 2022). Indeed, its genomic binding promotes local chromatin interactions with a profound impact on the ES transcriptional program (Showpnil et al., 2022). In agreement, EWS-FLI1 depletion readily reverses the oncogenic program toward mesenchymal differentiation (Flores and Grohar, 2021). Fine-tuned regulation of fusion TFs is crucial for their activity in ES, and the ETS-family TF, ETV6, is a selective dependency through regulation of EWS-FLI activity on chromatin (Lu et al., 2023). The EWS-WT1 fusion TF is expressed in DSRCT, and EWS-WT1 represses estrogen signaling and drives a proliferative and DNA damage response signature, suggesting potential mechanisms for chemotherapy resistance (Gedminas et al., 2020). DSRCT cells are dependent on the expression of EWS-WT1, as demonstrated by its selective silencing, with a consequent induction of apoptosis through the impact on downstream targets, FGFR4, JAK3, mTOR, PDGF, ERG, and TGFB.

In CSS, EWS-ATF1 has been described as a constitutive transcriptional activator, whose DNA binding activity is regulated by the phosphorylation of serine-266 on EWSR1 (Olsen and Hinrichs, 2001). Its expression is sufficient for sarcomagenesis in murine models and is enhanced by MYC expression (Panza et al., 2021). EWS-ATF1 exerts a potent chromatin regulatory activity by establishing enhancer networks that induces oncogenic signatures (Möller et al., 2022). Furthermore, the neoplastic behavior of the fusion protein is potentiated by the inhibition of p53-dependent transcriptional activation by sequestering the transcriptional coactivator CBP/p300 (Fujimura et al., 2001).

In ASPS, genome-wide binding analysis revealed that ASPSCR1-TFE3 acts as a strong transcriptional activator of target genes that contribute to neoplastic proliferation and survival (Table 1; Figure 3) (Kobos et al., 2013). Surprisingly, ASPSCR1-TFE3 expression is non-essential for *in vitro* cell growth but is necessary for *in vivo* tumorigenesis, directly regulating angiogenesis-associated clustered enhancers (Tanaka et al., 2023). Moreover, ASPSCR1-TFE3 activity enhances autophagy-related gene expression program, revealing a targetable vulnerability in this pathway (Barrott et al., 2019). In depth genomic analysis revealed that the segregase VCP/p97 is a necessary co-factor of ASPSCR1-TFE3, facilitating its required assembly to stimulate enhancer function, thus supporting the ASPSCR1-TFE3-dependent oncogenic signature (Pozner et al., 2024).

Umpolung is a German word, connoting a *polarity reversal*, and in undifferentiated round cell sarcoma (URCS), CIC-DUX4 reverses the natural “polarity” of CIC from a repressor to a global transcriptional activator (Table 1; Figure 3) (Hendrickson et al., 2024). CIC-DUX4 can activate key genes involved in sarcomagenesis including RAS and PI3K/AKT pathway genes (Hendrickson et al., 2024). The transcriptional functions of the umpolung chimera CIC-DUX4 can be targeted with translational approaches, including focusing on disruption of the P300 function interaction (Bosnakovski et al., 2021). P300 inhibitors can disrupt gene signatures driven from CIC-DUX4 activity in URCS, resulting in loss of tumor proliferation (Bosnakovski et al., 2021). Similar effects have been demonstrated through genetic approaches to inactivate P300 function in URCS, and also with targeted protein degradation approaches for P300/CBP (Bakaric et al., 2024).

### 1.4 ATP-dependent chromatin remodeling

It is of high interest that within the spectrum of fusion-driven sarcomas, there are varying degrees of mechanistic interrelatedness of the fusion oncoprotein with ATP-dependent remodeling complexes. A great set of examples is that mammalian SWI/SNF (BAF) complexes 1) directly incorporate the SS fusion oncoproteins (Kadoch and Crabtree, 2013), while 2) the ES fusions interact with SWI/SNF in the absence of chromatin but can participate in DNA binding site selection (Boulay et al., 2017), and 3) in RMS canonical BAF complexes interact with fusion oncoproteins supported through chromatin but not in the absence of chromatin (Laubscher et al., 2021). Each of these contexts provide a framework through which to examine fusion oncoprotein-remodeler interactions, which we hypothesize occur through intrinsically disordered domains (IDRs) of fusions across a constellation of childhood sarcomas. *Is the fusion incorporating into ATP-dependent remodeling complexes? Is the fusion biochemically interacting with SWI/SNF complexes through chromatin-supported induced proximity* (Stanton et al., 2018) *or rather are the IDRs sufficient to promote interactions even in the absence of chromatin scaffolding?* In addition to these key questions that are helping to shape the field, new impactful advances in understanding IDRs in a chromatin remodeling context (Patil et al., 2023) and advances in understanding how SWI/SNF subunits are functioning with modularity during the cell cycle (Zhu et al., 2023) and through dependencies on E3 ubiquitin ligases (Radko-Juettner et al., 2024) will catalyze a next-generation of studies to understand fusion-remodeler interactivity dynamics and stability. We describe key advances in studying SWI/SNF complexes in fusion-driven sarcomas, with an emphasis on these key questions.

In MLPS, the N-terminus of the fusion protein FUS-DDIT3 robustly interacts with three known types of SWI/SNF complexes, canonical BAF complex (cBAF), polybromo BAF complex (pBAF) and non‐canonical BAF (ncBAF) (Table 1; Figure 3) (Linden et al., 2022). These interactions alter the equilibria of antagonistic activity of SWI/SNF on Polycomb repressor complexes, affecting deposition of repressive histone modifications (Linden et al., 2019). Mechanistically, FUS-DDIT3 fusions contain Prion‐like domains (PLDs) that form dynamic phase condensates and drive phase separation in the nucleus. The engagement of PLDs on FUS-DDIT3 and PLDs in SWI/SNF assembled subunits mediate recruitment to the chromatin interface (Davis et al., 2021). Moreover, the SWI/SNF complex has been reported to mediate functional interactions between BRD4 and FUS-DDIT3 (Linden et al., 2022). Intriguingly, the fusion protein in MLPS hampers SWI/SNF-mediated activation of adipogenic enhancers by sequestering the TF CEBPB, resulting in a downregulated adipogenic signature and a concomitant upregulation of tumorigenic pathways (Zullow et al., 2022).

In FP-RMS, cBAF interacts with PAX3-FOXO1 through a DNA/chromatin interface (Laubscher et al., 2021). There is evidence of a hierarchical program in which CR TFs establish the enhancer landscape leading to cBAF recruitment at acetylated sites, thus fine-tuning CR TFs transcriptional activity. This stabilized RMS enhancer network interferes with the myogenic differentiation process locking RMS cells in a myoblastic-like state. Excitingly, the genetic depletion of SWI/SNF, or strategies for targeted protein degradation/inhibition lead a cell cycle arrest and the induction of myogenic enhancers (Laubscher et al., 2021). As an interesting parallel, in SS, SS18-SSX1 fusion protein has been described as a BAF subtype-specific subunit (Middeljans et al., 2012). The SS18-SSX1 fusion reversibly competes with SS18 wildtype for the assembly of BAF, driving the expression of pro-proliferative genes like SOX2 (Kadoch and Crabtree, 2013), which has been further studied with exciting new insights (Li et al., 2021). Interestingly, there is recent evidence that SS18-SSX can retarget BAF toward repressive chromatin domains leading to opposing the functional repression of silencing histone modifications (McBride et al., 2018; McBride et al., 2020; Tong et al., 2024). Furthermore, interactions of SS18-SSX1 with the histone demethylase KDM2B can drive neural-like gene expression signatures in SS (Banito et al., 2018).

### 1.5 Animal modeling for *in vivo* sarcoma etiology

The occurrence of oncogenic transformations within certain specific cell types has long been acknowledged. Indeed, patients with inherited mutations tend to develop cancers exclusively in particular organs (Miki et al., 1994), and the development of several cancers has been found to rely on the cell differentiation status (Barker et al., 2009). In this context, epigenetic regulatory mechanisms are essential in controlling differentiation and maintaining cell fate. We hypothesize that a combined interplay of the cancer genome and epigenome, dependent on cellular context, is necessary for cancer development. Moreover, sarcoma development and progression involve complex interactions between tumor cells, the tumor microenvironment, and host factors (Helman and Meltzer, 2003). Therefore, while cell lines have been extensively utilized as experimental models to study tumor biology and therapeutic responses, the need for *in vivo* models that possess higher complexity is critical.

The development and establishment of genetically engineered mouse models (GEMMs), patient-derived xenografts (PDXs), and syngeneic models, have offered the advantage of preserving tumor heterogeneity and interactions with the host immune system (Chuprin et al., 2023). These models recapitulate key features of sarcoma biology, including tumor initiation, progression, and response to therapy, offering opportunities to dissect these molecular mechanisms and identify critical drivers of sarcoma growth and metastasis. Moreover, these models are facilitating the translation of preclinical findings into clinical translation. However, open questions are at the forefront: *can animal models recapitulate clonal evolution/selection and genetic drift of human cancer cells? What should be done to maximize the translatability and etiological relevance for animal models, especially in the context of the immune system?* We focus on recent studies that encompasses the use of animal model in fusion-driven sarcomas.

In induced pluripotent stem cell models (iPSCs) derived from CSS, the inducible expression of the fusion oncogene EWS-ATF1 is sufficient for the formation of sarcomas in chimeric mice in a cell-type-dependent manner (Komura et al., 2019). Indeed, despite the expression of EWS-ATF1 in a high variety of tissues, secondary sarcomas preferentially occur in soft tissues in these CSS initiation models. This is due to EWS-ATF1-dependent activation of oncogene-induced senescence (OIS), that prevents cancer development in several somatic cell types, but not in soft tissues that give rise to sarcomas. EWS-ATF1 function selectively activates neural crest-related enhancers in peripheral nerves, identified as cells of origin for EWS-ATF1-induced sarcomas through a transgene driven cell-type specific promoter (Komura et al., 2019). In agreement, the epigenetic silencing of EWS-ATF1-bound enhancers restores OIS, highlighting the insurgence of premature senescence as a mechanism for the cell-type specificity in tumorigenesis. Once more, cell-type-related epigenetic regulation exerts the pivotal role for cancer cell fate.

As previously described, CIC-DUX4 in URCS behaves as a *de novo* transcriptional activator. Recently, it has been demonstrated that the spontaneous expression of the CIC-DUX fusion in a Cre-independent manner, induces sarcomas in chimeric mice (Hendrickson et al., 2024). Moreover, CIC haploinsufficiency, occurring in CIC-DUX4 sarcomas, is not required for the formation of tumors, thus supporting the key transforming role of the fusion oncogene in the complete penetrance observed. The development of a spontaneous-URCS mouse model also provides insight into the transcriptional signatures of the murine tumors suggesting a mesenchymal origin and resembling the human sarcomas (Hendrickson et al., 2024).

The development of an EHE mouse model in which the conditional expression of WWTR1-CAMTA1 in endothelial cells is paired with *CDKN2A* knockout, allows for the *in vivo* evaluation of the effects on tumorigenesis of *CDKN2A* loss, the most frequent secondary mutation in EHE cases (Seavey et al., 2023). Indeed, *CDKN2A* loss associates with an increased tumor growth and aggressiveness, recapitulating at histological and transcriptional levels the human disease (Seavey et al., 2023).

With the aim of characterizing whether novel fusions identified in RMS patients have oncogenic properties and the fundamental mechanisms involved, Zebrafish vertebrate models have been impactful (Kashi et al., 2015; Kent et al., 2024). For infantile and FP-RMS driven by VGLL2-NCOA2 and PAX3-FOXO1 fusions, respectively, fusion-oncogenes generate RMS tumors in fish that resemble the human ones (Kendall et al., 2018; Kent et al., 2023; Watson et al., 2023). Of note, while the human VGLL2-NCOA2 fusion induces tumors (Watson et al., 2023), PAX3-FOXO1 requires a background of tp53^M214K^ missense mutation, as a cooperative event (Kendall et al., 2018). As the Zebrafish RMS models histologically and molecularly recapitulate the human sarcoma, these systems can be useful to identify and evaluate therapeutic vulnerabilities and prognostic biomarkers. In agreement, ARF6 has been described as an actionable vulnerability for VGLL2-NCOA2 driven RMS (Watson et al., 2023) and HES3 as prognostic marker and a mediator of PAX3-FOXO1 tumorigenesis (Kendall et al., 2018). Exciting translational animal models have been developed over the course of several decades, allowing for mechanistic studies of sarcomagenesis, including diverse mouse syngeneic models (Keller et al., 2004a; Keller et al., 2004b; Searcy et al., 2023) and mouse xenograft models (Patel et al., 2022; Wei et al., 2022). We anticipate continued advances in the area of translational animal modeling for fusion-driven sarcomas to give insight into cell of origin, and the fundamental requirements for transformation and drug resistance.

## 2 Discussion

Several key questions emerge from our analyses of the literature. From the epigenetics and translational genomics studies in fusion-driven sarcomas, there are patterns that emerge in the combinatorial complexity of domain structures present in the fusions (Table 1; Figure 3). DNA-binding domains including Paired domain (PD) homeodomain (HD) chimeras emerge in RMS and URCS. In RMS and ASPS, bHLH TF chimeras are penetrant. In ES, CSS and URCS bZIP and ETS family TFs form fusion chimeras. There are also recurrent translocations of fragments of enzymes or TFs that incorporate into enzymes (RMS, SS, ASPS). With these highly recurrent patterns, exquisitely specific requirements are present for which fusions and which family members are transforming (i.e., there is no reported PAX4-FOXA2 as a transforming fusion in RMS, and there is no reported FOXO1-FLI in ES): why are the mechanisms that we have studied as a community so general, categorically? The major mechanistic categories of CRC, integrations with ATP-dependent chromatin remodeling, and influencing 3D chromatin architectural looping are fascinating and yet categorically general, while the requirements for fusion events that are transforming, and penetrant seem highly specific: *why is this?*

One possibility for the asymmetric dichotomy between the generality of the epigenetic mechanisms we are establishing as a community and the genetic specificity of the fusion partners is the technical difficulty in sequencing of repressive chromatin, both in the linear sense and the 3D architectural sense is so daunting. There is already strong evidence that H3K9me3 domains “drop out” of input samples in ChIP-seq (Becker et al., 2017) and as such we are limited in what we can “see” and measure outside of the open accessible regions. This technical challenge undoubtedly provides upward limitations in terms of the mechanisms we are able to observe and may contribute to the apparent generality of epigenetic mechanisms despite the exquisite specificity of fusion chimeras in sarcoma.

Furthermore, with exciting new technologies that are able to illuminate TF binding motifs within individual cells or subpopulations within a sarcoma, we will have exciting new higher complexity mechanisms from which to comprehensively elucidate chromatin state, hierarchical folding of chromatin, lineage plasticity, and examine disease etiology. Studies revealing that drug treatments can alter or select for specific subpopulations with a sarcoma continue to be impactful (Patel et al., 2022; Wei et al., 2022; Danielli et al., 2023a; Danielli et al., 2023b; DeMartino et al., 2023), and as we integrate increasing single cell epigenomic information into these studies, we will attain a greater understanding of how chemotherapy drugs interact with the epigenome in individual cells.

Finally, *does the generality of mechanistic interpretation for the fusion-driven sarcomas present convergent strategies for therapeutic targeting of fusion-driven sarcomas?* With increasing clinical development of SWI/SNF degraders and inhibitors, and exciting recent advances in direct targeting of transcription factors with chemically inducible proximity, we are at the horizon of next-generation therapeutic strategies, gaining extraordinary potency while avoiding both on-target and off-target toxicity, with the primary goal of rewiring the oncogenic circuitry of fusion-driven sarcomas. This is especially exciting given recent advances from the Cravatt lab in molecular targeting of the FOXA1 pioneer factors, which are closely related family members of the translocated FOXO1 in RMS (Won et al., 2024).

We anticipate further exciting advances at the intersectional space at the interface of translational genomics of fusion-driven sarcomas, chemically induced proximity strategies for molecular targeting of fusion events, and the mechanistic epigenetics to begin to provide more specific mechanisms including investigations of highly repressive chromatin regions as new technologies enable their investigation. The horizon for mechanistic advances leading to new paradigm for drug discovery and molecular targeting strategies for fusions will be transforming.

## Data Availability

The original contributions presented in the study are included in the article, in the form of our analysis of the scientific literature. No new datasets were curated as part of this project.
